# A Guide to Peer Coaching for Health Professions Educators

**DOI:** 10.1111/tct.70051

**Published:** 2025-02-26

**Authors:** James Fisher, Anna Ainsworth, Richard Thomson, Joanna Matthan, Vishna Devi V. Nadarajah, Yvonne Steinert

**Affiliations:** ^1^ School of Medicine Newcastle University Newcastle upon Tyne UK; ^2^ Newcastle University Medicine Malaysia Iskander Puteri Johor Malaysia; ^3^ Department of Family Medicine and Institute of Health Sciences Education, Faculty of Medicine and Health Sciences McGill University Montreal Canada

**Keywords:** peer coaching, peer observation, faculty development

## 1 Introduction

Despite a significant expansion in faculty development programmes for health professional educators [1], not all teachers have received education about teaching. Many teachers have, instead, learnt solely through teaching, with their educational practice being shaped by their prior experiences as both learners and as teachers.

Evaluating one's performance as an educator, with a view to improving one's teaching, is also challenging. Firstly, the shortcomings of self‐assessment are widely recognised and may be compounded by receiving incomplete feedback on one's actions [2]. Although seeking feedback from students is commonplace, there are limitations with this approach too. ‘Survey fatigue’ amongst students is recognised, and the consequences of this may be poor response rate [3], inaccurate evaluations [4] and responder bias, resulting in polarised opinions [5]. Issues may also arise with how practicable such feedback is. There is often a significant lag between the actual teaching moment and receiving feedback, meaning that the recollection of events may be limited. Furthermore, student feedback is invariably more student‐centric and not educator‐focussed.

Peer coaching is a faculty development approach whereby teachers receive personalised feedback on their teaching from colleagues, with the overarching aim of supporting their growth as educators. Although this approach is well suited to health professions education, and although there is evidence of its benefits, peer coaching is not universally adopted within institutions due to barriers, both perceived and real. In this Clinical Teachers' Toolbox article, we aim to encourage the uptake of peer coaching as a faculty development strategy by:

- Defining peer coaching and offering a structure for this faculty development activity.
- Describing a theoretical basis that underpins peer coaching.
- Outlining the benefits and potential barriers to peer coaching, alongside strategies to mitigate against these.
- Reflecting on how culture might influence implementation of peer coaching programs.

## 2 What Is Peer Coaching?

Peer coaching has been defined as ‘a collegial process whereby two faculty members voluntarily work together to improve or expand their approaches to teaching’ [6]. The term ‘peer’ captures a broad remit, with Gosling [7] defining peers as ‘colleagues from the same department, either of a similar status or (with) differentials of status, or … from another department’. A coach may be defined as ‘an experienced person who supports a learner or client in achieving a specific personal or professional goal by providing training and guidance, with a focus on relatively short term performance’ [8].

**FIGURE 1:**
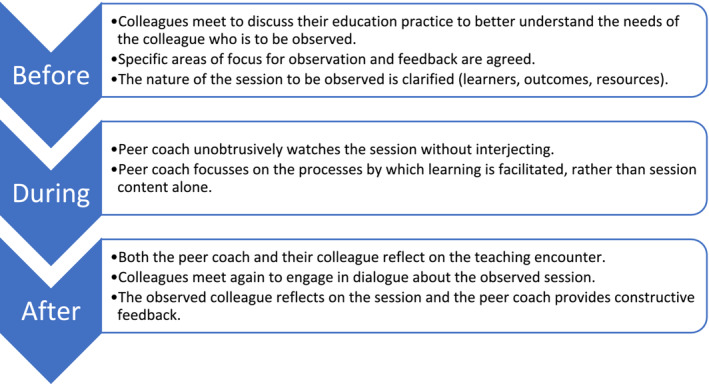
A three‐step approach to peer coaching.

Peer coaching requires a collaborative, stepwise approach, as outlined in Figure 1. Firstly, time is set aside before any observation for a dialogue between peer coaches and their colleagues. Colleagues' needs are explored and the focus of the upcoming observation, in terms of developmental goals, is agreed upon. The peer coach then unobtrusively observes their colleague undertaking the teaching session. Afterwards, colleagues meet again for further dialogue, where both share their observations and reflections, whilst working towards ways in which educational practice might be modified. In essence, peer coaching represents a tool for guided self‐reflection that is centred on individual performance, embedded in the workplace and underpinned by respectful relationships between colleagues.

“Peer coaching represents a tool for guided self‐reflection that is centred on individual performance, embedded in the workplace and underpinned by respectful relationships between colleagues.”

There is much diversity in how peer coaching initiatives can be structured. They may be formal or informal, voluntary or mandated. They may occur as a one‐off or instead run serially over a prolonged period. Participants may be organised into dyads or may instead form part of a larger group. The observation component may occur in real time or, instead, might employ video recordings to allow observation at a time of one's choosing.

When describing peer coaching, it is also necessary to acknowledge that other terminology is often employed. The term ‘peer observation’ is sometimes used, though this term's sole focus on observation risks neglecting the critical before and after elements that are required to translate observation into meaningful change to one's educational practice.

The term ‘mentoring’ is also sometimes employed to describe such initiatives. Although peer coaching and mentoring share similarities, there are differences between the two approaches. Mentoring has been defined as ‘a bidirectional relationship … which focusses on mentees professional growth’ [9]. Such relationships may be deeper, often focusing on personal life, work–life balance and career progression [10].

To conclude our definition of terms, Table 1 outlines what peer coaching is not and what it ought not to be.

**TABLE 1:** What peer coaching is not or ought not to be.

Peer coaching is not or ought not to be …	Justification
Solely an observation	As per Figure 1, it is critical that time is invested for colleague‐coach dialogue both before and after the observation
Reliant on the ‘expert‐learner’ model	The peer coach does not need to be an expert teacher, nor a topic expert, since the crux of observation relates to their colleague's ability to facilitate learning. Peer coaching is not about appraising a colleague's knowledge base
Summative	Peer coaching ought to be non‐evaluative, to cultivate a relatively safe space for honest, critical reflection on one's teaching and discussion of topic areas that may be challenging or even controversial
Prescriptive	Although the use of observation grids may provide a structure for note‐keeping during observations, rigidly sticking to these may risk drifting from the original focus of the observation, as identified at the pre‐observation meeting
Mandatory	Although this is debated, mandating observations risks the participant's engagement with peer coaching becoming extrinsically motivated, which may undermine the potential for meaningful learning about one's teaching
A one‐way process	Discussions before and after ought to be dialogues, not one‐way transmission of information from coach to colleague. The reciprocity of learning within peer coaching dyads is well recognised

## 3 A Theoretical Basis for Peer Coaching

**FIGURE 2:**
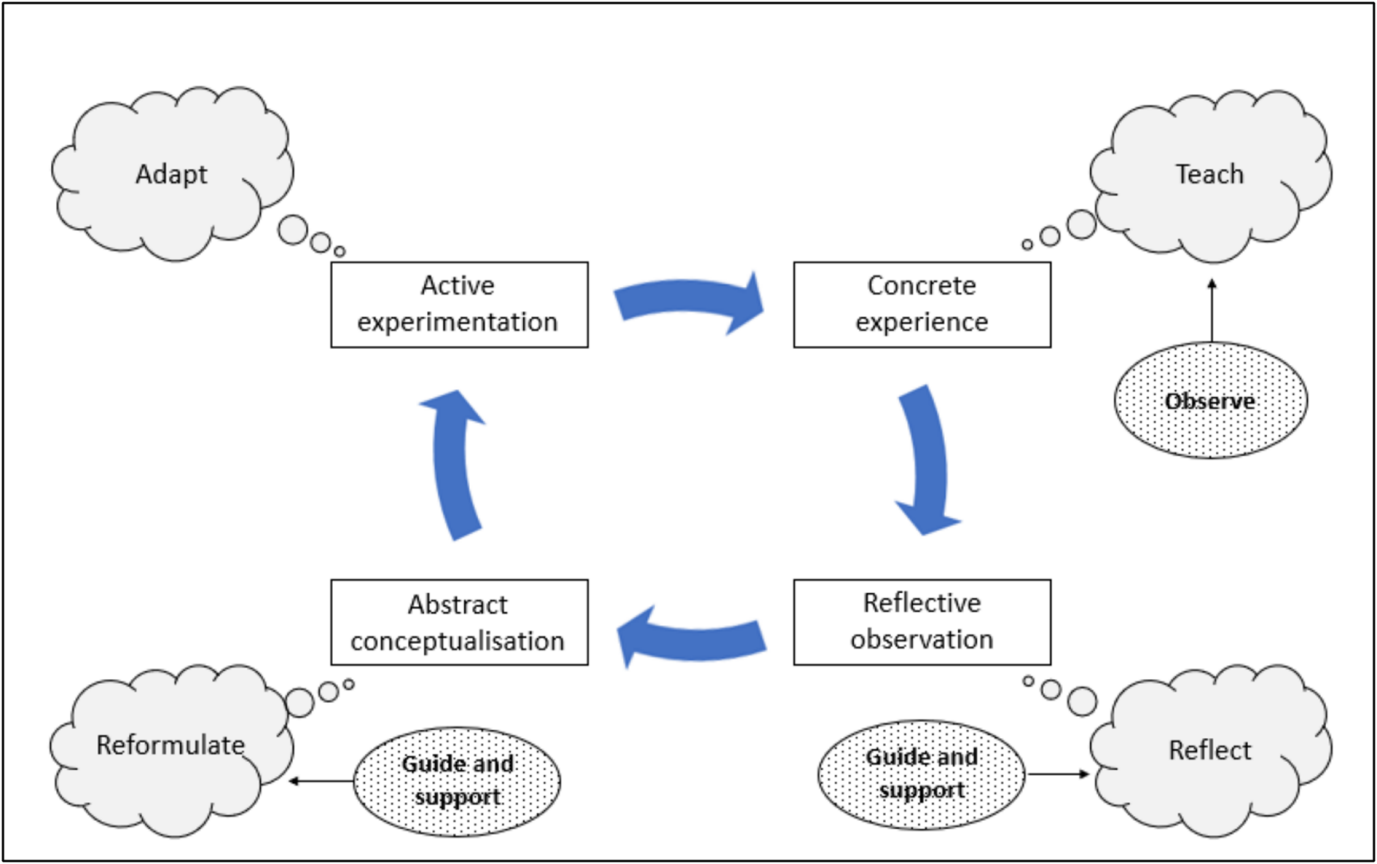
Kolb's experiential learning cycle [rectangles] as applied to peer coaching, with the actions of observed colleague [thought bubbles] and peer coach [ovals] depicted.

Seen through the lens of Kolb's experiential learning cycle [11], peer coaching may be seen as a means of supporting *active experimentation* before, and *reflective observation* after, a teaching episode, thereby creating new (in this case, pedagogical) knowledge through the ‘transformation of experience’ (Figure 2). By contrast, unobserved teaching is liable to err on the side of familiarity, to repeat errors, to miss opportunities and to stifle educator development. A well‐structured peer coaching cycle, like a good lesson plan, will encourage teachers to pause and reflect on the outcome they wish to achieve (e.g., active participation of the whole group of learners) and consider strategies to achieve it. This gives the coach a focus for their observations during the teaching episode, which will help their colleagues to reflect afterwards. If this gives rise to a sense of dissatisfaction or curiosity about alternative strategies, *abstract conceptualisation* may follow, for example, if the colleague decides to explore the literature on an alternative teaching strategy. This in turn may give rise to further active *experimentation* (with or without the coach observing) the next time the session is run.

“A well‐structured peer coaching cycle, like a good lesson plan, will encourage teachers to pause and reflect on the outcome they wish to achieve.”

**FIGURE 3:**
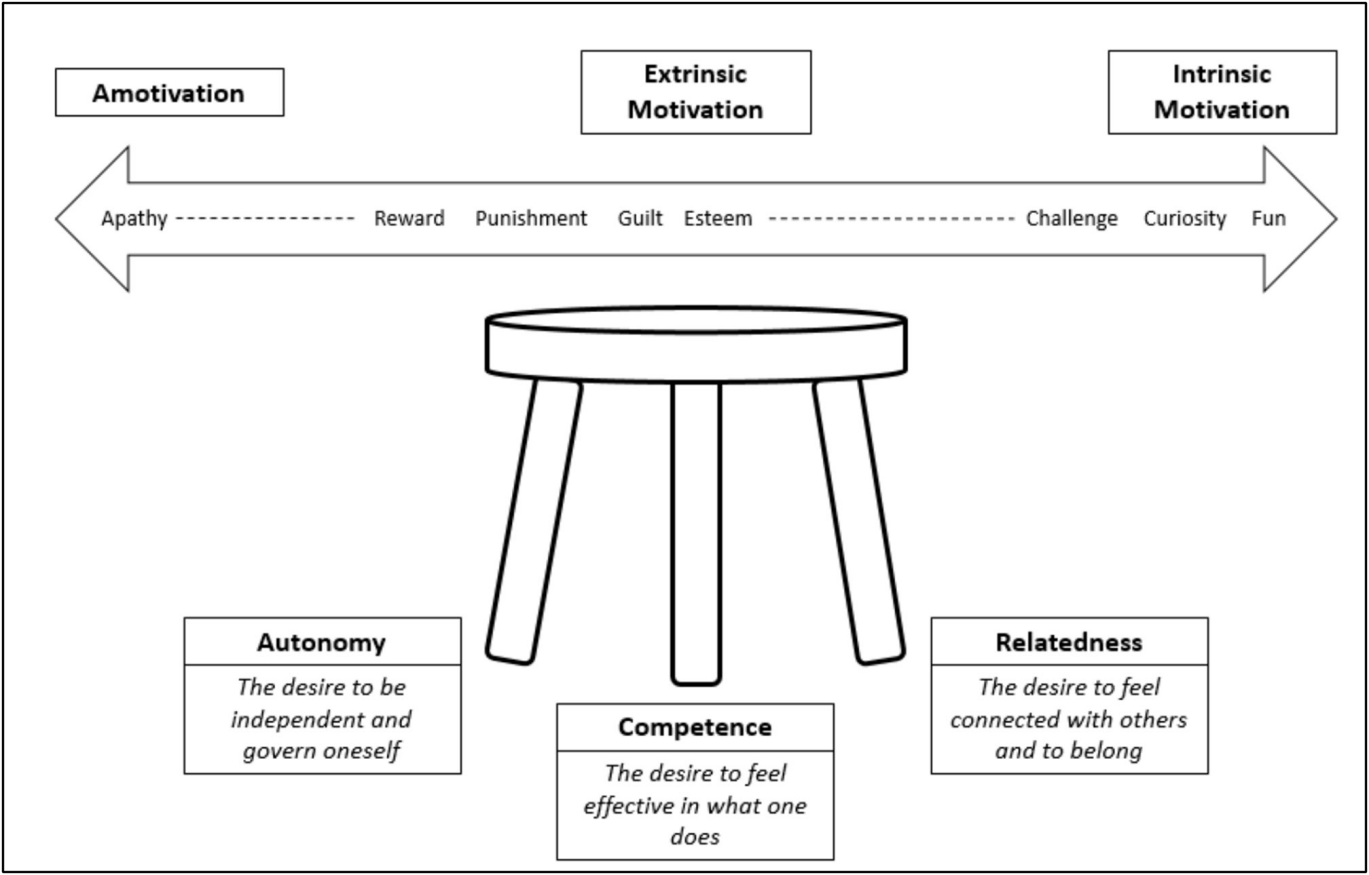
Self‐determination theory: a spectrum of motivation and innate, underlying psychological needs.

As we make clear throughout this paper, a challenge in implementing peer coaching is achieving buy‐in from colleagues, and it is helpful here to consider psychological theories of motivation. We find that self‐determination theory [12] helps us to explain peer coaching to colleagues and to gauge whether it has been effective (Figure 3). Done well, peer coaching engages the colleague's intrinsic motivation to do a good job (competence), to do it their way (autonomy) and to feel part of a teaching and learning community (relatedness). By contrast, if the process is perceived to be a pointless hoop‐jumping exercise or a covert surveillance operation conducted by the leadership team, colleagues may lose all desire to participate in the initiative (amotivation) or be driven to participate solely by an external locus of control (extrinsic motivation). Both amotivation and extrinsic motivation are associated with poorer performance and well‐being. If the goal is personal growth, one can readily appreciate that colleagues must therefore find intrinsic motivation to engage, and this principle can guide us on how to cultivate an environment that supports this.

## 4 The Benefits of Peer Coaching

Peer coaching builds on the development agendas of colleagues and establishes person‐centred growth as a foundational principle. This makes it a flexible tool that can be effective across experience and expertise levels [13]; indeed, peer coaching can benefit from diverse participants in a learning community, as the outcomes are co‐constructed. It is recognised that peer feedback can increase self‐worth for teachers [14], which, in turn, can promote workforce satisfaction and sustainability [15].

A bidirectional coaching relationship creates a supportive and collegiate approach, demanding equal participation between partners and mitigating power dynamics between colleagues [16, 17]. When both colleagues share a teaching context, they can develop a more nuanced understanding of the realities of the education setting, including its dynamism and its challenges.

Such collaboration fosters development via multiple pathways. A participant can develop new skills by receiving feedback on their own skills and through reflective, developmental dialogue with their peer coach. Peer coaching also brings reciprocal benefits to the peer coach, who, by observing and discussing their colleague's teaching, can develop their own teaching skills. As a collaborative process, peer coaching has the potential to strengthen existing relationships between colleagues or build new ones within the participants' community of practice [18]. Where institutional values align with peer coaching principles, reflective and developmental practice can become embedded. This may encourage teachers to adopt a more reflective, collaborative approach in their general educational practice [13] and may also facilitate cross‐pollination of good practice between teams and departments.

“Peer coaching also brings reciprocal benefits to the peer coach, who, by observing and discussing their colleague's teaching, can develop their own teaching skills.”

We acknowledge that despite these myriad benefits, there may be barriers to implementation of peer coaching initiatives. In Table 2, we share, from the perspective of the colleague being observed, these potential barriers alongside strategies we offer to mitigate against these.

**TABLE 2:** Potential barriers to peer coaching programmes and how they might be overcome.

‘This will not work because …’	How to mitigate against this
‘I do not have time for this’	Institutional‐level commitment to peer coaching initiatives ought to legitimise the use of time for this purpose; however, we acknowledge that educators are busy people, who often have a myriad of competing interests. The process of peer coaching need not, however, be time consuming. For example, depending on the area of focus for observation that is identified within discussion, the peer observation component itself may not necessarily require the entire session to be viewed
‘There'll be too much paperwork’	The steps and rubrics employed within peer coaching should be simple and easy to follow. Employing technology to record these may make their completion more efficient
‘I feel uneasy about being observed’	Observational anxiety with peer coaching is widespread and understandable, and a lack of psychological safety within peer dyads risks undermining their educational value. Rather than ignoring this, we suggest leaders acknowledge its existence and recognise that there is no ‘one size fits all’ strategy. For example, some teachers may find observation by their peers to be threatening, whereas others may find a familiar face to be less intimidating than a staff member from a different department. Giving staff members agency when it comes to selecting their peer coach may help reduce this sense of unease
‘I'll teach differently if I'm being watched’	Observer placement within teaching sessions ought to be as unobtrusive as possible, seeking to avoid the eye line of their colleague where feasible. Fostering a collaborative relationship within the pre‐observation discussion, with a focus on support and development rather than critique, may go some way to minimise the impact of being observed
‘My institution must have an agenda for doing this’	A ‘top‐down approach’ where peer coaching is mandated and seemingly foisted on staff without clarity of purpose risks staff interpreting the scheme as a mechanism of quality control, with potential negative consequences for their career. Leaders should therefore take the time to clearly articulate the rationale for the initiative so that peer coaching is instead conceptualised as a tool for personal development
‘The observer being there will interfere with students' learning’	Whereas the observer ought to be as unobtrusive as possible in the session, the peer coach should be introduced to the students at the start of the session. Their role should be clearly explained so that students understand that they are there to observe their teacher and not them
‘No one else seems to be doing this’	Hearing real‐life accounts from staff who have engaged with, and benefitted from, peer coaching may help challenge preconceptions and raise the profile of peer coaching within the department
‘I'm unsure if the peer coach is trained to give useful feedback’	Observer selection and training requirements ought to be communicated to all stakeholders. There should be opportunities for peers to have the role of being observed and to observe, to benefit from both perspectives
‘It's hard to synchronise suitable times’	Administrative support to assist with identification of mutually convenient times may help, as might the use of freely accessible online software whereby colleagues can indicate their availability
‘I have not received any training for this’	Peer coaching schemes ought not to be parachuted onto staff without any accompanying training. Providing faculty development to help staff acquire and hone the skills needed for them to observe, and then facilitate discussions, will increase the likelihood of meaningful engagement amongst staff members
‘My peer coach is from a very different culture and does not understand the teaching environment I work in’	Discussion within the pre‐observation meeting provides an ideal opportunity for the peer coach to learn about the educational milieu in which their teaching colleague works. Making cultural differences a valid topic for discussion offers the potential for rich bilateral learning with the observer potentially then able to offer more nuanced, context‐specific observations
‘I'm concerned about being asked a question that I cannot answer. What if I get something wrong, or say something stupid?’	No teaching session is perfect. This should be acknowledged within the pre‐observation discussion, where it should also be stated that the observer's role is to watch teaching process, rather than scrutinise sessions for content missteps or slips of the tongue
‘I do not see the value in this exercise’	Encourage leaders to share their personal reflections of peer coaching to demonstrate its potential impact on teaching. This may encourage those staff members who are sceptical to engage with the initiative regardless, as after completing a discussion–observation–discussion cycle, the value of peer observation will become apparent
‘My audience might think I am not a good educator as I need to be observed’	The purpose of peer coaching programmes needs to be clearly communicated to students. The practice of receiving feedback to enhance competencies is part of reflective practice, and peer coaching is an excellent way for health professionals and faculty to role model this to students
‘The chemistry between my coach and I means this will not work’	Significant personality clashes may render some peer coaching dyads incompatible, but we suggest that this would be infrequently encountered. Giving teachers agency to identify the colleague that they would like to link up with may help in a situation where conflict exists between staff members

## 5 The Impact of Culture

For educators who are seeking to introduce, or refine, a peer coaching programme in their institution, the influence of ‘departmental culture’ is likely to be at the forefront of their thinking. Faculty development, and health professions education more generally, is provided within a diverse range of cultural contexts. Yet, it is recognised that culture is somewhat of an amorphous notion that is variably and infrequently defined, thus making it challenging to determine which elements of culture are relevant to faculty development initiatives such as peer coaching [19]. We choose to define culture using the ‘three lens’ approach described by Watling et al. [20], whose novel theorisation offers a helpful framework for reflecting on how peer coaching initiatives might be embedded.

The first lens Watling describes is the ‘organisational perspective’, where organisational culture is the product of the shared assumptions, beliefs and values that characterise a setting. This is typically considered in retrospect and is often construed as being a barrier to change, rather than as a resource that can itself be a catalyst for change. For example, in the cultural context of hierarchical societies, great trust and respect is placed in authority figures, which can manifest in deference to, and more guarded dialogue with, colleagues with perceived seniority [21]. Hofstede's cultural dimension theory also recognises this phenomenon. Hofstede's six categories that define culture include ‘power distance’ [22]. In countries where this is large, the culture is one where there is great respect for, and deference to, rank, authority and experience—in essence, ‘subordinates expect to be told what to do’ [22]. For peer coaching to be effective, open discourse, where vulnerabilities can be shared, is crucial. One might conclude that a culture with large power distance would therefore be incompatible with peer coaching. Yet, as Watling suggests, this cultural factor may provide potential for change, as the power of positive role‐modelling is amplified in such a cultural setting [23], making senior colleagues, who are invested in peer coaching, particularly powerful agents for change. Yet, the potency of this cultural phenomenon, and the extent to which it is in entrenched, must not be underestimated when planning peer coaching initiatives.

Second, Watling refers to the ‘identity perspective’, where identity and culture become intertwined. Through this lens, culture is about how people make sense of themselves within a given community or setting. Teacher identity is recognised as an important determinant of excellence in education and something that departments ought to strive to nurture through faculty development initiatives [24]. A teacher's identity is not a passive possession; instead, it is dynamic, actively colouring what a teacher values, how they practice and how they develop. Considering peer coaching, an educator whose teacher identity is secure and comfortable may be inclined to perceive peer observation as an opportunity to develop. But an educator whose teacher identity feels more insecure may perceive the same opportunity as a chance to ‘fail’. Legitimising discussion about teacher identity within peer coaching initiatives is therefore important and should form part of any accompanying training or briefing.

Finally, Watling's ‘practice perspective’, which, perhaps counterintuitively, eschews culture and instead focuses on practice, that is, what happens, and how is it enacted by people and things? Through this lens, culture does not exist in terms of ‘some unseen force directing people and their practice’ [20]; there are instead only actions that happen in time and space. For example, consider the educator seeking to implement peer coaching who is encountering resistance to doing so within their department. Rather than drawing on assumptions and interpretations of culture, the practice perspective would encourage this educator to examine the multitude of elements that influence peer coaching practice (e.g., identifying a peer colleague, timetabling and the physical spaces where observation/discussion occurs). A deliberate, analytical examination of all the elements that inform practice may reveal flaws that can be remedied, rather than erroneous assumptions about the individuals or the culture perpetuating sub‐optimal practice.

We close this discussion around culture with an acknowledgement that though the three lenses were considered separately, they are in practice, often co‐existent, either colliding or complementing depending on the educational milieu. Furthermore, aiming for a ‘one size fits all’ standard for peer coaching is not our aspiration. We have instead sought to moderate our guidance through cultural humility. This approach acknowledges that there are likely to be a variety of effective, contextually appropriate approaches to peer coaching globally [25] and that there is much to be learnt through international collaboration and bidirectional learning [19].

“There are likely to be a variety of effective, contextually appropriate approaches to peer coaching globally and that there is much to be learnt through international collaboration and bidirectional learning.”

## 6 Conclusion

In this toolbox article, we offer a definition and a structure for embedding peer coaching within a health professions education context. The potential benefits of implementing peer coaching are outlined whilst acknowledging the barriers that exist to doing so and offering mitigation strategies against these. Accessible summaries of relevant educational theory are presented, seeking to illuminate the pedagogy that underpins the three‐step approach to peer coaching that is suggested. Theory is also used to offer explanations for variable engagement with peer coaching programmes. The impact of the local educational milieu on peer coaching schemes is further considered through discussion about culture.

## Author Contributions

**James Fisher:** conceptualization, writing – original draft, writing – review and editing, project administration, visualization. **Anna Ainsworth:** conceptualization, writing – original draft, writing – review and editing, visualization. **Richard Thomson:** conceptualization, writing – original draft, writing – review and editing, visualization. **Joanna Matthan:** conceptualization, writing – review and editing, visualization. **Vishna Devi Nadarajah V:** conceptualization, writing – review and editing, visualization. **Yvonne Steinert:** conceptualization, writing – review and editing, visualization.

## Conflicts of Interest

The authors declare no conflicts of interest.

## Data Availability

The authors have nothing to report.
